# Analysis of *BRCA*1 and *BRCA*2 mutations in Brazilian breast cancer patients with positive family history

**DOI:** 10.1590/S1516-31802005000400007

**Published:** 2005-07-07

**Authors:** Rozany Mucha Dufloth, Sílvia Carvalho, Juliana Karina Heinrich, Júlia Yoriko Shinzato, César Cabello dos Santos, Luiz Carlos Zeferino, Fernando Schmitt

**Keywords:** Breast neoplasms, Hereditary diseases, BRCA1 gene, BRCA2 gene, Base sequence, Câncer da mama, Doenças hereditárias, Gene BRCA1, Gene BRCA2, Seqüências do DNA

## Abstract

**CONTEXT AND OBJECTIVE:**

*BRCA*1 and *BRCA*2 are the two principal hereditary breast cancer susceptibility genes, and the prevalence of their mutations among Brazilian women is unknown. The objective was to detect *BRCA*1 and *BRCA*2 mutations in Brazilian patients with breast cancer, so as to establish genetic profiles.

**DESIGN AND SETTING:**

Cross-sectional study, in Centro de Atenção Integral à Saúde da Mulher, Universidade Estadual de Campinas, Brazil, and Institute of Pathology and Molecular Immunology, University of Porto, Portugal.

**METHODS:**

Thirty-one breast cancer patients with positive family history (criteria from the Breast Cancer Linkage Consortium) were studied, and genomic DNA was extracted from peripheral blood. Single-strand conformation polymorphism was used for the analysis of exons 2, 3, 5, and 20 of *BRCA1*. Cases showing PCR products with abnormal bands were sequenced. Exon 11 of *BRCA1* and exons 10 and 11 of *BRCA2* were directly sequenced in both directions.

**RESULTS:**

Four mutations were detected: one in *BRCA*1 and three in *BRCA*2. The *BRCA*1 mutation is a frameshift located at codon 1756 of exon 20: 5382 ins C. Two *BRCA*2 mutations were nonsense mutations located at exon 11: S2219X and the other was an unclassified variant located at exon 11: C1290Y.

**CONCLUSION:**

The *BRCA*1 or *BRCA*2 mutation prevalence found among women with breast cancer and such family history was 13% (4/31). Larger studies are needed to establish the significance of *BRCA* mutations among Brazilian women and the prevalence of specific mutations.

## RESUMO

**CONTEXTO E OBJETIVO:**

*BRCA*1 e *BRCA*2 são os dois principais genes de susceptibilidade ao câncer de mama hereditário e a prevalência de mutações nestes genes não é conhecida em mulheres brasileiras. O objetivo do estudo foi detectar mutações de *BRCA*1 e *BRCA*2, contribuindo para estabelecer um perfil dos carcinomas de mama hereditários na população brasileira.

**TIPO DE ESTUDO E LOCAL:**

Estudo transversal, no Centro de Atenção Integral à Saúde da Mulher, Universidade Estadual de Campinas, Brasil, e no Instituto de Patologia e Imunologia Molecular da Universidade do Porto, Portugal.

**MÉTODOS:**

31 pacientes com carcinoma de mama e história familiar (critérios do *Breast Cancer Linkage Consortium*), foram estudadas e tiveram DNA extraído do sangue periférico. *Single strand conformation polymorphism* (SSCP) foi empregado para analisar os *exons* 2, 3, 5, e 20 do *BRCA*1. Aqueles casos que mostraram produtos de reação da polimerase em cadeia (PCR) com bandas anormais foram seqüenciados. Entretanto, o *exon* 11 do *BRCA*1 e *exons* 10 e 11 do *BRCA*2 foram diretamente para o seqüenciamento.

**RESULTADOS:**

Foram identificadas quatro mutações, sendo uma mutação no *BRCA*1 e três no *BRCA*2. A mutação no *BRCA*1 é do tipo *frameshift,* no *exon* 20: 5382 insC. Duas mutações encontradas no *BRCA*2 são do tipo *nonsense* localizadas no *exon* 11: S2219X e uma do tipo *unclassified variant* localizada no *exon* 11: C1290Y.

**CONCLUSÃO:**

A prevalência de mutações de *BRCA*1 e *BRCA*2 encontradas para mulheres com câncer de mama e história familiar de câncer de mama foi de 13% (4/31). Estudos mais amplos são necessários para estabelecer o significado dessas mutações na população brasileira.

## INTRODUCTION

Epidemiological studies have revealed several risk factors associated with increased susceptibility to breast cancer. Among these, familial history is one of the most important. Five to 10% of breast tumors are believed to be hereditary,^1,2^ and about 30% of young women who develop breast cancer are likely to show a genetic pattern of predisposition to the disease. This hypothesis is confirmed if these women go on to develop bilateral carcinomas associated with other neoplasias such as carcinoma of the ovary or colon, or if they show an autosomally dominant inheritance pattern.^3,4^

In this context, and particularly in highrisk families, the most important tumor suppressor genes associated with breast cancer are *BRCA*1 and *BRCA*2. Women who carry *BRCA*1 mutations have a probability of about 80% for developing breast cancer, and 40 to 60% for developing ovarian cancer during their lifetime.^5^ Moreover, these women have a 65% probability for developing a second breast carcinoma if they reach the age of 70.^6-8^ Women who carry *BRCA*2 mutations have a likelihood of developing breast cancer of about 85%.^6,8,9^

*BRCA*1 is a tumor suppressor gene mapped to position q21 of chromosome 17. It is made up of more than 80 kb, distributed in 22 exons, coding for a protein of 1,863 amino acids.^10,11^ Exon 11 comprises 60% of the gene, making it the main target for mutation detection. *BRCA*2 is another tumor suppressor gene mapping to locus 13q12, comprising 10.4 kb and organized in 27 exons that code for a protein of 3,418 amino acids.^12,13^ Mutations in both *BRCA*1 and *BRCA*2 are spread throughout the entire gene. More than 600 mutations of *BRCA*1 and 450 mutations of *BRCA*2 have been described, according to the Breast Cancer Information Core website (BIC).^14^

There are several methods for identifying *BRCA* mutations. The choice of method depends on the resources available in the laboratory and on the existence of any previously identified mutation in the family or in the patient's ethnic group, although identification should always be confirmed by sequencing.

The aim of this study was to detect *BRCA*1 and *BRCA*2 mutations in a group of Brazilian patients with breast cancer, in an attempt to establish a genetic profile for this population. This information would facilitate *BRCA*1 and *BRCA*2 mutational screening in the Brazilian population. Moreover, the detection of mutations in the patient's family allows identification of individuals at high risk, who are then able to seek genetic counseling.

## METHODS

### Informed consent

Clinical information, pathology reports, slides, paraffin blocks and blood samples were obtained with the informed consent of patients under the guidelines and approval of the Research Ethics Committee of the School of Medical Sciences, Unicamp, and the National Committee for Ethics in Research (Conep).

### Patient selection

Thirty women and one man with a diagnosis of carcinoma of the breast and a positive family history of breast cancer, who were receiving treatment at the Breast Cancer Outpatient Department of Centro de Atenção Integral à Saúde da Mulher, Universidade Estadual de Campinas (CAISM/Unicamp), Brazil, were identified and invited to participate in this study. The criteria for the identification of individuals at high risk were based on the Breast Cancer Linkage Consortium^15^ criteria: early onset (at less than 45 years of age) and/or bilaterality; more than three cases of breast cancer and more than one case of ovarian cancer in the family; more than two first-degree relatives involved; and male breast cancer.

### DNA extraction and mutation detection

Genomic DNA was extracted from peripheral blood using the phenol:chloroform method, following a standard protocol.^16^ We performed molecular analysis on exons 2, 3, 5, 11 and 20 of the *BRCA1* gene and exons 10 and 11 of the *BRCA2* gene. For this study, we used single-strand conformation polymorphism and direct sequencing methods.

### Single-strand conformation polymorphism

Single-strand conformation polymorphism (SSCP) was used for the analysis of exons 2, 3, 5, and 20 of the *BRCA1*gene. The primers used for these exons are described in Table 1. The polymerase chain reaction (PCR) was carried out using 250 ng of DNA, 1 x PCR buffer with 1.5 mM of MgCl (Amersham Biosciences, Piscataway, New Jersey), 200 µM of each dNTP (Amersham Biosciences, as above), 10 ρmol of each primer, 1U of *Taq* DNA polymerase (Amersham Biosciences, as above) at a final volume of 25 µl. The PCR conditions were 96° C for five minutes, then 35 cycles of 30 seconds at 96° C, 30 seconds at the annealing temperature of the primer, 1 minute at 72° C followed by one cycle at 72° C for 10 minutes. For the SSCP analysis, the PCR reaction products were diluted in 1:1 loading buffer (95% formamide, 0.05% bromophenol blue and 0.05% xylene cyanol), and denatured at 98° C for 10 minutes. Electrophoresis of the denatured PCR products was carried out in non-denaturing 0.8 X detection enhancement gels (BMA, Rockland, Maine) at 170 W for 16 hours. In all the cases in which SSCP analysis showed an abnormal electrophoretic pattern, the sample was sequenced in both directions.

**Table 1:** Primer list used for the analysis of exons 2, 3, 5, 11 and 20 of the *BRCA*1 gene

				Annealing temperature
Exon 2	F:	GAA GTT GTC ATT TTA TAA ACC TTT		57° C
	R:	TGT CTT TTC TTC CCT AGT ATG T		57° C
Exon 3	F:	TCC TGA CAG AGC AGA CAT TTA		53° C
	R:	TTG GAT TTT CGT TCT CAC TTA		53° C
Exon 5	F:	CTC TTA AGG GCA GTT GTC AG		58° C
	R:	TTC CTA CTG TGG TTG CTT CC		58° C
Exon 20	F:	ATA TGA CGT GTC TGC TCC AC		57° C
	R:	GGG AAT CCA AAT TAC ACA GC		57° C
Exon 11	1	F:	CCA AGG TGT ATG AAG TAT GT	57° C
		R:	GAT CAG CAT TCA GAT CTA CC	57° C
	2	F:	CTC ACT AAA GAC AGA ATG	56° C
		R:	CTT TCT GAA TGC TGC TAT	56° C
	3	F:	CAG AAA CTG CCA TGC TTC AGA	56° C
		R:	AGG CTT GCC TTC TTC CGA TA	56° C
	4	F:	GTT CAC TCC AAA TCA GTA GAG AG	56° C
		R:	CAG CTT TGC TTT TGA AGG CAG	56° C
	5	F:	CCT AAC CCA ATA GAA TCA CTC G	56° C
		R:	GAA CCA GGT GCA TTT GTT AAC TTC	56° C
	6	F:	CAG CGA TAC TTT CCC AGA GC	56° C
		R:	GTC CCT TGG GGT TTT CAA A	56° C
	7	F:	CTG GAA GTT AGC ACT CTA GG	56° C
		R:	GTT GCA CAT TCC TCT TCT GC	56° C
	8	F:	CCG TTT TCA AAT CCA GGA AA	56° C
		R:	TGA TGG GAA AAA GTG GTG GT	56° C
	9	F:	GAG GCA ACG AAA CTG GAC TCA	56° C
		R:	CTC AGG TTG CAA AAC CCC TA	56° C
	10	F:	AAC AGA GGG CCA AAA TTG AA	56° C
		R:	GGG TGA AAG GGC TAG GAC TC	56° C
	11	F:	AAA GCG TCC AGA AAG GAG AGC	56° C
		R:	GCC TTT GCC AAT ATT ACC TGG	56° C
	12	F:	CAT TGA AGA ATA GCT TAA ATG	56° C
		R:	CCT GGT TCC AAT ACC TAA GTT	56° C

### Direct sequencing

PCR products with abnormal bands in the SSCP pattern were sequenced. In addition, in each patient sample, exon 10 of the *BRCA2* gene and 11 of both *BRCA* genes were directly sequenced in both directions. Exon 11 of the *BRCA1* gene was divided into 12 overlapping fragments. Exon 10 of the *BRCA2* gene was divided into four overlapping fragments and exon 11 was divided into 16 overlapping fragments (primer sequences are described in Tables 1 and 2). Sequencing was performed by the dideoxy chain termination method, using Big Dye^®^ technology (Applied Biosystems, Foster City, California). The sequencing primers were the same as those used for PCR. The cycling conditions were as follows: 96° C for five minutes, then 35 cycles of 30 seconds at 94° C, 30 seconds at 51° C, four minutes at 60° C, followed by one 10-minute cycle at 60° C. The products were purified using an MgCl_2_/ethanol-based protocol and run on an ABI 3100 sequencer (Applied Biosystems, as above). The results were analyzed using the 3100 data collection software. The sequencing was repeated twice for each sample to rule out the possibility of PCR fidelity artifacts, and was carried out in both directions.

**Table 2:** Primer list used for the analysis of exons 10 and 11 of the *BRCA*2 gene

				Annealing temperature
Exon 10	1	F	GTG CTT CTG TTT TAT ACT TT	52° C
		R	CCA TTA GAT TCA AAT GTA G	52° C
	2	F	CTC ATT TGT ATC TGA AGT GG	56° C
		R	GAG AGA TGA AGA GCA GCA TC	56° C
	3	F	GCC ATT AAA TGA GGA AAC AG	56° C
		R	GAT AAT GGA AGC TGG CCA GC	56° C
	4	F	CTG TTT GCT CAC AGA AGG AG	52° C
		R	GAT TCA GGT ACC TCT GTC	52° C
Exon 11	1	F	AGT GAA TGT GAT TGA TGG TAC	54° C
		R	CAT GCT GCA GCC AAG ACC TCT	54° C
	2	F	GAA GGA CAG TGT GAA AAT G	54° C
		R	CCT TTC TTG AAG GTG ATG C	54° C
	3	F	AAG ATG TAT GTG CTT TAA ATG	54° C
		R	CTC CTC TGC AAG AAC ATA AAC	54° C
	4	F	AGA CAC AGG TGA TAA ACA AG	54° C
		R	CAA GGT ATT TAC AAT TTC AA	54° C
	5	F	GCT CTC TGA ACA TAA CAT TAA G	58° C
		R	CAT TAT GAC ATG AAG ATC AG	58° C
	6	F	TAT CTT AAA GAC CAC TTC TG	54° C
		R	TGA AAC AAC AGA ATC ATG AC	54° C
	7	F	CTT CTG CAG AGG TAC ATC	54° C
		R	CAG TAA ATA GCA AGT CCg	54° C
	8	F	TTT GAT GGC AGT GAT TCA AG	54° C
		R	CTT ATG TCA GAA TGT AAT TC	54° C
	9	F	ATC AGA AAC CAG AAG AAT TG	54° C
		R	ATC TCA ATG GTC TCA CAT GC	54° C
	10	F	CAG AGA GGC CTG TAA AGA C	54° C
		R	GAA GTC TGA CTC ACA GAA G	54° C
	11	F	TGA AAA TTC AGC CTT AGC	54° C
		R	GCA TCT TTT ACA TTG GAT	54° C
	12	F	GTA TTG AGC CAG TAT TGA AG	54° C
		R	TGC CTC GTA ACA ACC TGC CAT	54° C
	13	F	GTT TCA GTA AAG TAA TTA AG	54° C
		R	AGA TTT TCC ACT TGC TGT GC	54° C
	14	F	AAG TCA GTC TCA TCT GCA A	54° C
		R	GAA ACT TGC TTT CCA CTT G	54° C
	15	F	CAT CTG CTT TCT CTG GAT TTA	56° C
		R	ATG TTC TCA ACA AGT GAC ACT	56° C
	16	F	ATG TTG AAG GTG GTT CTT CAG	56° C
		R	GTG ATT GGC AAC ACG AAA GG	56° C

## RESULTS

In four cases (13%), changes in the normal sequence of *BRCA*1 and *BRCA*2 genes were identified: one of these mutations occurred in the *BRCA*1 gene and the other three in the *BRCA*2 gene.

**Figure 1:**
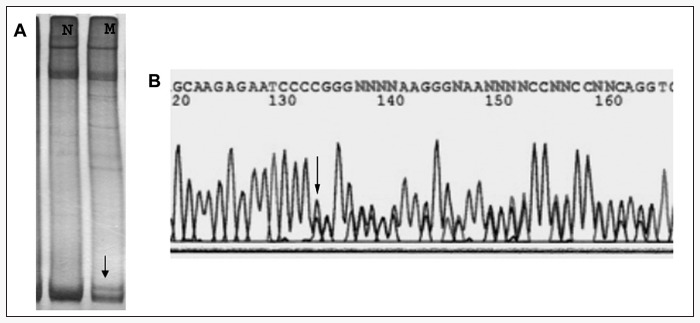
(A) Single-strand conformation polymorphism pattern of exon 20 of the *BRCA*1 gene of wild-type DNA sample (N) and mutated DNA (M) sample; (B) sequencing pattern of the sample with aberrant band in the SSCP gel. This corresponds to the 5382 ins C mutation. This mutation was identified in a patient who developed breast cancer at the age of 33 and with a first-degree relative with the disease.

The alteration in exon 20 of *BRCA*1 was found in a patient who developed breast cancer at the age of 33, and who has a first- degree relative who had also developed the disease. Furthermore, the anatomopathological profile of the carcinoma presented several characteristics that are usually associated with hereditary carcinoma, such as c-erbB2 expression, negativity for hormonal receptors, and high histological grade. Migration alterations were found by SSCP and, after sequencing, a mutation was found in nucleotide 5382, codon 1756 of *BRCA*1 exon 20. This mutation is referred to as 5382 ins C, according to the BIC database. It is a *frameshift mutation*that originates in a premature stop codon (STOP 1829) and leads to the formation of a truncated protein (Figure 1).

**Figure 2:**
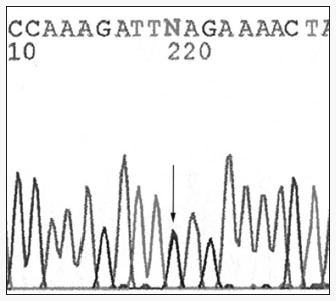
Sequencing pattern showing a nonsense mutation (change of a serine to a stop codon) in nucleotide 6884 of exon 11 of the *BRCA*2 gene. This mutation is designated S2219X. This mutation was identified in two patients, both who developed the disease before 45 years and with second-degree relatives with breast cancer.

The *BRCA*2 mutations were detected in two patients who developed the disease before the age of 45 years and who have at least two second-degree relatives with breast carcinoma. After sequencing, the mutation was localized in exon 11 of *BRCA*. This is a *nonsense* mutation originating from a stop codon in nucleotide 6885, referred to as S2219X according to the BIC database (Figure 2).

**Figure 3:**
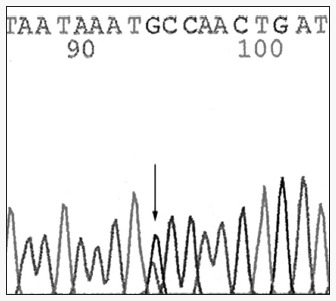
Sequencing pattern showing a mutation (change of a cysteine to a tyrosine) in nucleotide 4097 of exon 11 of the *BRCA*2 gene. This mutation is still an unclassified variant, and was identified in a patient who developed breast cancer at the age of 29, with no relatives with cancer.

The other *BRCA*2 mutation was also found in exon 11 of *BRCA*2 in a patient who developed the disease at the early age of 29 years, and who had no relatives affected by breast cancer. The mutation is still an unclassified *variant* according to the BIC database and is referred to as C1290Y, so it is impossible to affirm that it is not a pathogenic mutation (Figure 3).

## DISCUSSION

Cloning of the *BRCA*1^11^ and *BRCA*2^12^ genes, the major genes known to confer high risk of breast and ovarian cancer, has resulted in the characterization of a large number of mutations in both genes (Breast Cancer Information Core database). Apart from specific ethnic groups, there is no predominant mutation to account for the majority of cases of inherited breast cancer. In some places, recurrent mutations have been described which facilitate the search for mutations in both genes. In spite of the high prevalence of breast cancer in the Brazilian population, there has not been any systematic study of *BRCA*1 and *BRCA*2 mutations among breast cancer patients with a family history of the disease.

In the present study, we analyzed 31 breast cancer patients selected according to the criteria adopted by the Breast Cancer Linkage Consortium^15^ for hereditary breast cancer. We detected four mutations (13%), one in *BRCA*1 and three in the *BRCA*2 gene. The *BRCA*1 mutation is a frameshift mutation located at codon 1756 of exon 20: 5382 ins C. This mutation has been previously described in Ashkenazi Jews and is clearly associated with an increased risk of breast cancer.^17^ The woman with this mutation in the present study developed breast cancer at 33 years of age and she has a first-degree relative with breast cancer. Considering the prevalence of descendants of Ashkenazi Jews in the Brazilian population, it is not surprising to find this mutation in our group of patients.^18^

All three *BRCA*2 mutations found in our study are novel mutations. There were two nonsense mutations located at exon 11: S2219X and one unclassified variant located at exon 11: C1290Y. The S2219X mutation was recently described in a Spanish population from Castilla-Leon.^19^ In that study, this mutation was considered to be a novel mutation in the Spanish population. As far as we know, this is the first time that this mutation has been described in the Brazilian population. Although the ancestry of these two patients was specifically investigated, neither of them was found to have Spanish ancestors. Both developed breast cancer before reaching 45 years of age and both had two second-degree relatives with breast cancer.

The method used in the present study, namely direct sequencing, is an expensive technique, but it is the best technique for detecting less frequent mutations and unclassified mutations, as we found in our study sample. Thus, in addition to selecting the patients according to clinical-pathological criteria,^20^ we should also study specific populations in order to detect recurrent mutations. This may allow us to establish a more cost-effective mutational analysis for this population.

In the present study, most of the mutations detected were novel mutations, which indicates that the mutational screening restricted to prevalent mutations that has previously been reported cannot be recommended in our population. The Brazilian population, like the population of the United States, is ethnically mixed, and founder mutations are therefore rare or even absent. Many European mutations have been observed in the United States and Canada, reflecting European migration to North America. Similarly, in many cases, Central and South American families can trace their origins to the period of Spanish or Portuguese colonization. However, although a previous study in another South American country also demonstrated mutations related to the Ashkenazi Jews,^21^ previous studies carried out by our group among a Portuguese population^22^ and a Spanish population from Galicia^23^ failed to show the mutations that were detected in the present study in the Brazilian population. Further studies are necessary for establishing the relevance of all of these alterations in our population.

This is the first study to investigate *BRCA*1 and *BRCA*2 mutations among Brazilian patients with breast cancer. The identification of *BRCA*1 and *BRCA*2 mutations is relevant for establishing preventive strategies for women with breast cancer and *BRCA* mutations, in order to prevent contralateral breast tumors and ovarian tumors. In addition, the detection of mutations in the patient's family allows identification of individuals at high risk, who can then seek genetic counseling.

## CONCLUSION

The prevalence of the *BRCA*1 or *BRCA*2 mutation found in this study among women with breast cancer and a family history of breast cancer was 13% (4/31). Large studies are necessary for establishing the significance of the *BRCA* mutation among Brazilian women and the prevalence of specific mutations. Knowledge of the spectrum of mutations together with their geographical distribution in Brazil is necessary for establishing an effective detection strategy.
